# Variation in Seed Germination of 134 Common Species on the Eastern Tibetan Plateau: Phylogenetic, Life History and Environmental Correlates

**DOI:** 10.1371/journal.pone.0098601

**Published:** 2014-06-03

**Authors:** Jing Xu, Wenlong Li, Chunhui Zhang, Wei Liu, Guozhen Du

**Affiliations:** 1 State Key Laboratory of Grassland and Agroecosystems, School of Life Science, Lanzhou University, Lanzhou, P.R. China; 2 State Key Laboratory of Grassland and Agroecosystems, School of Pastoral Agriculture Science and Technology, Lanzhou University, Lanzhou, P.R. China; Institute of Botany, Chinese Academy of Sciences, China

## Abstract

Seed germination is a crucial stage in the life history of a species because it represents the pathway from adult to offspring, and it can affect the distribution and abundance of species in communities. In this study, we examined the effects of phylogenetic, life history and environmental factors on seed germination of 134 common species from an alpine/subalpine meadow on the eastern Tibetan Plateau. In one-way ANOVAs, phylogenetic groups (at or above order) explained 13.0% and 25.9% of the variance in germination percentage and mean germination time, respectively; life history attributes, such as seed size, dispersal mode, explained 3.7%, 2.1% of the variance in germination percentage and 6.3%, 8.7% of the variance in mean germination time, respectively; the environmental factors temperature and habitat explained 4.7%, 1.0% of the variance in germination percentage and 13.5%, 1.7% of the variance in mean germination time, respectively. Our results demonstrated that elevated temperature would lead to a significant increase in germination percentage and an accelerated germination. Multi-factorial ANOVAs showed that the three major factors contributing to differences in germination percentage and mean germination time in this alpine/subalpine meadow were phylogenetic attributes, temperature and seed size (explained 10.5%, 4.7% and 1.4% of the variance in germination percentage independently, respectively; and explained 14.9%, 13.5% and 2.7% of the variance in mean germination time independently, respectively). In addition, there were strong associations between phylogenetic group and life history attributes, and between life history attributes and environmental factors. Therefore, germination variation are constrained mainly by phylogenetic inertia in a community, and seed germination variation correlated with phylogeny is also associated with life history attributes, suggesting a role of niche adaptation in the conservation of germination variation within lineages. Meanwhile, selection can maintain the association between germination behavior and the environmental conditions within a lineage.

## Introduction

Seed germination is one of the most extensively researched areas in plant biology [1]. The timing and level of germination strongly affect a plant's recruitment success and may consequently have implications for species migration [2]. According to recent studies, it is reasonable to expect that, seed germination could be affected by phylogeny [3], [4], life history attributes such as seed size [5], [6], seed dispersal [7], life form [8], [9] and environmental signals [10], [11]. Among the many environmental factors, temperature is perhaps more important in determining suitable conditions for seedling establishment, while other factors are germination triggers or cues [12].

However, there are three problems in these studies. Firstly, most studies have measured the effects of one variable at a time, ignoring the possibility that correlations among several phylogenetic and life history variables may confound the effects of any single variable on seed germination. For example, seed size is related with seed dispersal [13], habitat [14] and growth form [15], [16]. Consequently, to assess the role of natural selection on seed germination at the community level, we should take into account as many variables as possible when measuring the effect of any single variable. Nevertheless, only a few studies focus on the effect of phylogenetic, life history and environmental correlates on seed germination. For example, Wang et al. (2009) investigated seed germination of 69 arid/semi-arid zone species [17]. Secondly, although it is important to predict future distributions of species [18] and the germination study of seeds collected from one community at the same time may provide important information to understand the dynamics of a community, very few studies have been addressed to test seed germination in an alpine/subalpine community by combining phylogenetic analysis. Thirdly, temperature is predicted to increase with climate change [19] and the warming is much more intense in mountainous and high-elevation regions than at low altitude [20], [21]. To alpine/subalpine plants, which are expected to be affected more by climate warming [22], the effects of temperature on germination have hardly been studied from a community perspective. Therefore, we expect to advance our understanding on how phylogenetic, life history and environmental factors to regulate seed germination in an alpine/subalpine meadow community.

In this study, we chose 134 common species collected from the alpine/subalpine meadow on the eastern Tibetan Plateau, and the following questions were addressed (a) Whether differences in seed germination among species from the same community are related to phylogeny, life history traits, and/or environmental factors? (b) What proportion of germination variation among species could be attributed to the species' phylogenetic background, life history attributes, and environmental conditions? (c) Does a higher germination temperature affect germination of alpine/subalpine plants, and if so, how?

## Materials and Methods

### Study site

The study area is located on the eastern Tibetan Plateau (101°-103°E, 34°-35.70°N). The altitude ranges from 2800 to 4200 m, and the climate is cold Humid-Alpine with a mean annual precipitation (snow and rainfall) of 620 mm. Mean annual temperature is 2–3°C, and mean January and July temperatures are −10.7°C and 11.7°C, respectively. There is an average of 270 frost days per year. The grassland is dominated by native monocotyledons such as species of Poaceae and Cyperaceae and by native dicotyledons such as species of Ranunculaceae, Polygonaceae, Saxifragaceae, Asteraceae, Scrophulariaceae, Gentianaceae, and Fabaceae.

### Seed collecting and germination tests

In this study, seeds of 134 common species (Table S1) were collected from private grasslands of the study site from July to October in 2009 after we obtained permission from the grasslands owners, our studies did not involve endangered or protected species, and all the germination experiments were carried out in our own laboratory. For each species, seeds were collected at the beginning of their dispersal period to ensure they were mature. For a single species, seeds were collected from one site but from more than 20 plants. Seeds were air dried after collection, cleaned and stored at room temperature (approximately 15°C). For every species, three replicates of 100 air-dried seeds were randomly selected and weighed, and average mass per seed was calculated.

Before the germination experiments, the viability of seeds of each species was tested with tetrazolium chloride [23]. Only species with a seed viability of ≥99% were used in the germination experiments. The germination experiments were started on the middle of March (starting season of germination in the study area) in 2010. Seeds were placed in Petri dishes (9 cm diameter) on double layers of moistened filter paper, and then placed in growth chambers (Conviron E15 Growth Chamber, Controlled Environments Ltd., Winnipeg, Canada) under five different incubation treatments: 5/15°C (12:12 h) was simulated natural conditions prevailing in the soil at 5 cm depth in April and May in the study area (control treatment). 5/20°C (12:12 h), 5/25°C (12:12 h), 10/20°C (12:12 h) and 10/25°C (12:12 h) were simulated temperature increase. This experiment used three replications of 50 seeds of each species per temperature treatment. Seeds were kept saturated with distilled water. Dishes were randomized, stacked, and placed in temperature chamber. Seeds were incubated under darkness and a relative humidity of about 70% for 60 days. The seeds were checked for germination daily, at which time they were exposed to light for a few minutes. Thus, any light requirement for seed germination was fulfilled during these exposures [10]. The visible protrusion of the radicle was the criterion for germination. Germinated seeds were removed from the Petri dishes at each counting. At the end of each experiment, the remaining ungerminated seeds were tested for viability by staining with tetrazolium chloride, and the proportion of unviable seeds was calculated.

### Statistical analysis

Germination percentage (GP) was defined as the proportion of seed germinated, and seed mortality was defined as the proportion of unviable seeds tested with tetrazolium chloride after germination experiments. Mean germination time (GT) was estimated as follows: GT  =  ∑(*G_i_*×*i*)/∑(*G*_i_), where *i* is the day of germination, counted since the day of sowing, and *G_i_* is the number of seeds germinated on day *i* [24]. Three species that did not germinate at the end of the experiments were not included in this calculation, i.e., 131 species were used in GT analysis (Table S1).

First, a composite phylogeny of 134 species was constructed with Phylomatic version 3 (http://phylodiversity.net/phylomatic/) based on the angiosperm megatree (R20120829) [25]. Branch lengths were made proportional to time using the ‘bladj’ function in the program Phylocom 4.0 [26] and divergence time was estimated based on fossil data [27], [28]. To test the robustness of our results to uncertainties associated with branch length estimates, we also ran our analyses on the same composite tree, but with branch lengths set to 1. The resulting phylogenetic tree was used for subsequent analyses. We tested for the existence of phylogenetic signal by estimating Pagel's *λ* for GP and GT using “fitContinuous” functions in the R package “geiger” version 1.99-3 [29], using a maximum likelihood framework to estimate the parameter *λ*, which can vary from 0 (no influence of phylogeny) to 1 (strong phylogenetic influence) [30].

Then, one-way, two-way and multi-factorial ANOVAs were used to determine the effects of phylogeny and various life history (i.e., seed size, dispersal mode, life form, onset of flowering, duration of flowering) and environmental attributes (i.e., temperature and habitat) on GP and GT. One-way ANOVAs measured the effects of each factor on the variance of GP and GT across all other variables; two-way ANOVAs were conducted to detect significant interactions and associations between factors; multi-factorial ANOVAs tested the effect of each class variable independent of the others. We conducted a series of ANOVAs which include all variables but one (incomplete model). When each of these ANOVAs was compared to the ANOVA including all variables (complete model), the difference between the proportion of the total sum of squares (ss) explained by the complete model (its *R^2^*) and the *R^2^* of the incomplete model represented the proportion of the total ss explained by the deleted class variable [3]. Besides, multi-factorial ANOVAs corroborate associations between factors suggested by the two-way ANOVAs. If in the complete ANOVA, a given class variable had a lower *R^2^* value than in the incomplete ANOVA from which a different variable had been deleted, the increase in the *R^2^* value of the first variable would be due to an association (or correlation) or strong interaction with the second variable [3]. To carry out the statistical analysis, we grouped 134 species according to the following categories:

*1. Phylogenetic group.* Each of the 134 species was assigned to a family and an order according to Angiosperm Phylogeny Group III [31] (Table S1). When comparing the GP and GT between families, families containing more than seven species were chosen.

*2. Life form. *Species were grouped into two classes: annual and perennial.

*3. Dispersal mode. *Species were classified into four groups according to the morphological features of their seeds [4]: unassisted, ant-dispersed, adhesion-dispersed and wind-dispersed.

*4. Seed size. *The mean seed size of each species was assigned to 1 of 5 seed size classes according to Baker [32]: 0.032–0.099 mg, 0.100–0.315 mg, 0.316–0.999 mg, 1.000–3.161 mg, 3.162–9.999 mg.

*5. Onset of flowering. *Each species was grouped based on the Flora of China [33] and field observation records: early, flowering begins in May; middle, flowering begins in June; or late, flowering begins in July and August.

*6. Duration of flowering. *Each species was grouped based on the Flora of China [33] and field observation records: short, flowering duration of 1 month; median, flowering duration of 2–3 months; or long, flowering duration of ≥4 months.

*7. Temperature. *Based on temperatures occurring in the species' habitats, rising trend and the optimum alternating temperature regime of the most species [10], 5/15°C (control treatment), 5/20°C, 5/25°C, 10/20°C and 10/25°C were chosen.

*8. Habitat. *The habitats were classified into three categories: bottomland, north slope and south slope.

Because the data were unbalanced, all ANOVAs were conducted using GLM procedure of SPSS 13.0. The type III sum of squares was used to establish the significance level of each effect. In addition, both GP and mortality were arcsine square root transformed, and GT were log-transformed to improve normality and stabilize variances.

## Results

### Phylogenetic correlates

The results indicated that phylogenetic signals (*λ*) for GP and GT were 0.53 and 0.75, respectively. Both λ values were significantly different from 0 (*χ^2^* tests, both *P*<0.001).

**Figure 1:**
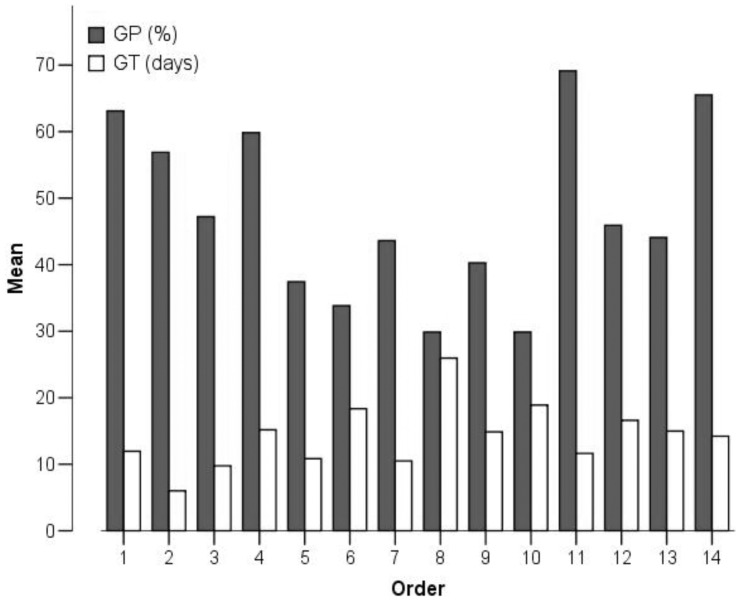
Germination percentage (GP) and mean germination time (GT) of seeds from 14 orders. 1 = Asterales, 2 = Brassicales, 3 = Caryophyllales, 4 = Ericales, 5 = Fabales, 6 = Gentianales, 7 = Lamiales, 8 = Liliales, 9 = Malpighiales, 10 = Myrtales, 11 = Poales, 12 = Ranunculales, 13 = Rosales, 14 = Saxifragales.

**Figure 2:**
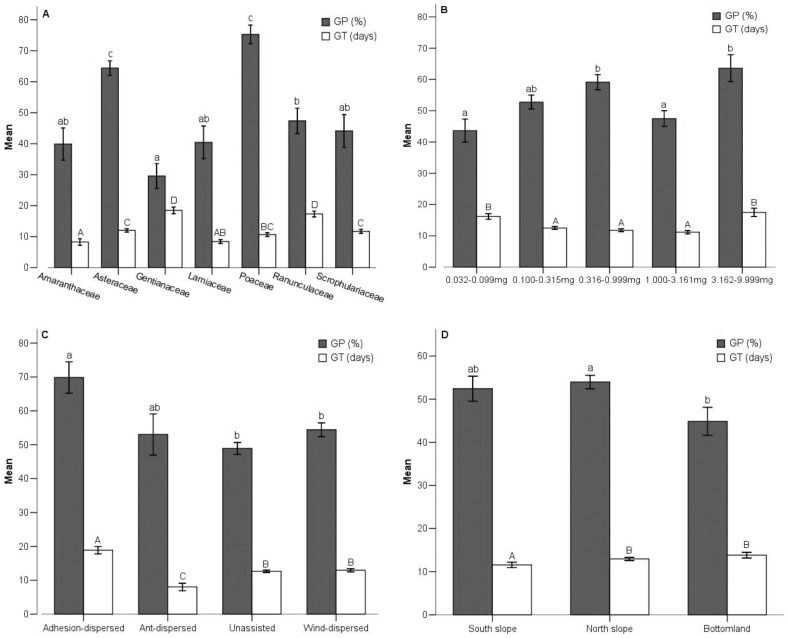
Germination percentage (GP) and mean germination time (GT) in different taxonomic groups. (A) GP and GT of seeds from seven families; (B) GP and GT of seeds from five seed size groups; (C) GP and GT of seeds from four dispersal mode groups; (D) GP and GT of seeds from three habitats. Bars (mean±SE) that do not share a letter represent significantly different values at *P*<0.05 level (Turkey multiple comparison test). Different lowercase letters and capital letters indicate significant difference of GP and GT, respectively.

One-way ANOVAs indicated that both GP and GT were significantly different among taxa (Figure 1, 2A), and order membership could account for 13.0% of the variance in GP and 25.9% of the variance in GT (Table 1). Thus, the majority of seed germination variation took the form of variation within orders.

**Table 1:** Results of one-way ANOVAs showing effect of phylogeny, life form, seed size, dispersal mode, onset of flowering, duration of flowering, temperature and habitat on final germination percentage (GP) and mean germination time (GT) among 134 species, *R^2^* is the proportion of variance explained by each factor.

Source of variation	Germination percentage (GP)				Mean germination time (GT)		
	df	*F*	*P*	*R^2^*	*F*	*P*	*R^2^*
Phylogenetic group	13	7.560	<0.001	0.130	17.219	<0.001	0.259
Life form	1	0.061	0.804	<0.001	52.508	<0.001	0.074
Seed size	4	6.318	<0.001	0.037	10.900	<0.001	0.063
Dispersal mode	3	4.821	0.003	0.021	20.579	<0.001	0.087
Onset of flowering	2	4.147	0.016	0.012	0.528	0.590	0.002
Duration of flowering	2	2.042	0.131	0.006	6.872	0.001	0.021
Temperature	4	8.123	<0.001	0.047	25.258	<0.001	0.135
Habitat	2	3.392	0.034	0.010	5.778	0.003	0.017

Two-way ANOVAs yielded significant interaction terms for phylogenetic relatedness when analyzed in combination with life history attributes, such as seed size, life form (Table 2).

**Table 2:** Results of two-way ANOVAs showing the independent effects of one of two main factors that have significant effects in one-way ANOVAs and interaction effects on germination percentage (GP) and mean germination time (GT) due to phylogenetic group (P), life form (LF), seed size (SS), dispersal mode (DM), onset of flowering (OF), duration of flowering (DF), temperature (T) and habitat (H). *R^2^* is the proportion of variance explained by each factor (Only significant interaction terms are shown).

Source of variation	Effect of A				Effect of B				Effect of A×B			
A/B	df	*F*	*P*	*R^2^*	df	*F*	*P*	*R^2^*	df	*F*	*P*	*R^2^*
Germination percentage (GP)												
P/SS	13	6.441	<0.001	0.117	4	5.989	<0.001	0.037	23	1.637	0.031	0.056
P/OF	13	5.523	<0.001	0.101	2	1.021	0.361	0.003	12	3.853	<0.001	0.067
SS/H	4	8.663	<0.001	0.050	2	2.457	0.086	0.007	7	3.330	0.002	0.034
DM/OF	3	6.616	<0.001	0.029	2	6.390	0.002	0.019	5	5.530	<0.001	0.040
OF/H	2	1.313	0.270	0.004	2	7.443	0.001	0.022	4	4.218	0.002	0.025
Germination time (GT)												
P/LF	13	12.561	<0.001	0.205	1	19.899	<0.001	0.030	7	3.073	0.003	0.033
P/SS	13	13.267	<0.001	0.219	4	8.064	<0.001	0.050	21	2.624	<0.001	0.082
P/DM	13	9.412	<0.001	0.163	3	8.222	<0.001	0.038	9	4.867	<0.001	0.065
P/DF	13	14.506	<0.001	0.231	2	3.452	0.032	0.011	10	2.748	0.003	0.042
LF/SS	1	46.552	<0.001	0.067	4	12.122	<0.001	0.070	4	3.406	0.009	0.021
LF/DM	1	21.975	<0.001	0.033	3	5.963	0.001	0.027	3	7.007	<0.001	0.031
LF/H	1	56.872	<0.001	0.081	2	7.038	0.001	0.021	2	4.679	0.010	0.014
SS/DM	4	4.475	0.001	0.027	3	12.428	<0.001	0.055	6	6.015	<0.001	0.053
SS/DF	4	10.883	<0.001	0.063	2	0.391	0.676	0.001	5	4.258	0.001	0.032
SS/H	4	8.120	<0.001	0.048	2	6.110	0.002	0.019	7	3.119	0.003	0.033
DM/DF	3	21.719	<0.001	0.092	2	1.875	0.154	0.006	4	2.670	0.031	0.016
DM/H	3	17.752	<0.001	0.076	2	0.444	0.641	0.001	5	5.343	<0.001	0.040
DF/H	2	16.325	<0.001	0.048	2	5.107	0.006	0.016	4	6.262	<0.001	0.037

The multi-factorial ANOVAs suggested the variance in GP and GT explained by order independently were 10.5% and 14.9%, respectively (Table 3, 4). In the multi-factorial ANOVAs of GT, the *R^2^* of life form and dispersal mode increased from 4.6% to 6.8% and from 2.8% to 5.7% respectively when phylogenetic group was deleted from the model, which suggested there were associations between phylogenetic group and life form and between phylogenetic group and dispersal mode (Table 4).

**Table 3:** Multi-factorial ANOVAs for the independent effects of each main factor and their associations.

Source of variation	df	*F*	*P*	*R^2^*	df	*F*	*P*	*R^2^*
Complete model					Phylogenetic group removed			
Phylogenetic group	13	6.559	<0.001	0.118				
Life form	1	0.130	0.719	<0.001	1	0.444	0.506	0.001
Seed size	4	2.869	0.022	0.018	4	4.127	0.003	0.025
Dispersal mode	3	1.707	0.164	0.008	3	2.037	0.107	0.009
Onset of flowering	2	2.797	0.062	0.009	2	3.727	0.025	0.011
Duration of flowering	2	0.756	0.470	0.002	2	1.065	0.345	0.003
Temperature	4	9.520	<0.001	0.056	4	8.569	<0.001	0.050
Habitat	2	1.049	0.351	0.003	2	2.132	0.119	0.007
Model	31	5.789	<0.001	0.220	18	4.710	<0.001	0.115
Seed size removed					Dispersal mode removed			
Phylogenetic group	13	7.006	<0.001	0.124	13	6.666	<0.001	0.119
Life form	1	0.043	0.835	<0.001	1	0.207	0.649	<0.001
Seed size					4	3.565	0.007	0.022
Dispersal mode	3	2.619	0.050	0.012				
Onset of flowering	2	2.792	0.062	0.009	2	3.822	0.022	0.012
Duration of flowering	2	1.895	0.151	0.006	2	0.669	0.513	0.002
Temperature	4	9.411	<0.001	0.055	4	9.489	<0.001	0.056
Habitat	2	1.870	0.155	0.006	2	0.889	0.412	0.003
Model	27	6.150	<0.001	0.206	28	6.206	<0.001	0.213
Onset of flowering removed					Temperature removed			
Phylogenetic group	13	6.728	<0.001	0.120	13	6.229	<0.001	0.112
Life form	1	0.083	0.773	<0.001	1	0.123	0.726	<0.001
Seed size	4	2.867	0.023	0.018	4	2.725	0.029	0.017
Dispersal mode	3	2.384	0.068	0.011	3	1.621	0.183	0.008
Onset of flowering					2	2.656	0.071	0.008
Duration of flowering	2	0.970	0.380	0.003	2	0.718	0.488	0.002
Temperature	4	9.467	<0.001	0.056				
Habitat	2	1.506	0.223	0.005	2	0.996	0.370	0.003
Model	29	5.962	<0.001	0.213	27	4.973	<0.001	0.173
Habitat removed								
Phylogenetic group	13	6.761	<0.001	0.121				
Life form	1	0.220	0.639	<0.001				
Seed size	4	3.290	0.011	0.020				
Dispersal mode	3	1.602	0.188	0.007				
Onset of flowering	2	3.263	0.039	0.010				
Duration of flowering	2	0.431	0.650	0.001				
Temperature	4	9.519	<0.001	0.056				
Habitat								
Model	29	6.115	<0.001	0.217				

Dependent variable is germination percentage (GP). For each main factor, *R^2^* is the proportion of the Type III sum of squares attributed to the main effect. The proportion of the variance explained by each class variable independent of others examined by the difference between the *R^2^* of the complete model and the *R^2^* of the model from which this class variable has been deleted.

**Table 4:** Multi-factorial ANOVAs for the independent effects of each main factor and their associations.

Source of variation	df	*F*	*P*	*R^2^*	df	*F*	*P*	*R^2^*
Complete model					Phylogenetic group removed			
Phylogenetic group	13	14.066	<0.001	0.227				
Life form	1	29.940	<0.001	0.046	1	46.681	<0.001	0.068
Seed size	4	8.135	<0.001	0.050	4	8.694	<0.001	0.052
Dispersal mode	3	6.078	<0.001	0.028	3	12.851	<0.001	0.057
Onset of flowering	2	4.495	0.012	0.014	2	1.452	0.235	0.005
Duration of flowering	2	2.885	0.057	0.009	2	4.616	0.010	0.014
Temperature	4	41.292	<0.001	0.210	4	32.589	<0.001	0.170
Habitat	2	5.897	0.003	0.019	2	6.077	0.002	0.019
Model	31	19.510	<0.001	0.493	18	18.501	<0.001	0.344
Life form removed					Seed size removed			
Phylogenetic group	13	15.590	<0.001	0.245	13	14.302	<0.001	0.229
Life form					1	24.116	<0.001	0.037
Seed size	4	6.645	<0.001	0.041				
Dispersal mode	3	5.971	0.001	0.028	3	11.765	<0.001	0.053
Onset of flowering	2	3.841	0.022	0.012	2	3.693	0.025	0.012
Duration of flowering	2	5.336	0.005	0.017	2	3.348	0.036	0.011
Temperature	4	39.462	<0.001	0.202	4	39.494	<0.001	0.201
Habitat	2	9.046	<0.001	0.028	2	6.247	0.002	0.020
Model	30	18.313	<0.001	0.468	27	20.272	<0.001	0.466
Dispersal mode removed					Duration of flowering removed			
Phylogenetic group	13	16.031	<0.001	0.250	13	14.435	<0.001	0.231
Life form	1	29.728	<0.001	0.045	1	35.127	<0.001	0.053
Seed size	4	12.499	<0.001	0.074	4	8.393	<0.001	0.051
Dispersal mode					3	5.862	0.001	0.027
Onset of flowering	2	2.914	0.055	0.009	2	5.171	0.006	0.016
Duration of flowering	2	2.553	0.079	0.008				
Temperature	4	40.311	<0.001	0.205	4	41.045	<0.001	0.208
Habitat	2	6.457	0.002	0.020	2	4.917	0.008	0.015
Model	28	20.452	<0.001	0.478	29	20.533	<0.001	0.488
Temperature removed					Habitat removed			
Phylogenetic group	13	11.190	<0.001	0.188	13	14.122	<0.001	0.227
Life form	1	23.818	<0.001	0.037	1	36.505	<0.001	0.055
Seed size	4	6.472	<0.001	0.040	4	8.322	<0.001	0.051
Dispersal mode	3	4.835	0.002	0.023	3	6.454	<0.001	0.030
Onset of flowering	2	3.576	0.029	0.011	2	5.893	0.003	0.019
Duration of flowering	2	2.295	0.102	0.007	2	1.904	0.150	0.006
Temperature					4	40.655	<0.001	0.206
Habitat	2	4.691	0.009	0.015				
Model	27	12.953	<0.001	0.358	29	20.133	<0.001	0.483

Dependent variable is mean germination time (GT). For each main factor, *R^2^* is the proportion of the Type III sum of squares attributed to the main effect. The proportion of the variance explained by each class variable independent of others examined by the difference between the *R^2^* of the complete model and the *R^2^* of the model from which this class variable has been deleted.

### Life history correlates

#### Life form

One-way ANOVAs indicated that the impact of life form on GP was not statistically significant, whereas the effect of life form on GT was notable (Table 1). The GT of annuals (11.03±0.50 days, mean±SE, hereafter) showed earlier than that of perennials (13.76±0.33 days). Two-way ANOVAs showed significant interaction terms for life form when analyzed in combination with phylogeny, seed size, dispersal mode and habitat (Table 2). The multi-factorial ANOVAs suggested life form accounted for 2.5% of the variance in GT independently (Table 4).

#### Seed size

Generally, species with heavier seeds (seed size ranged from 3.162 mg to 9.999 mg) had the highest GP (63.58±4.34%) and the most delayed GT (17.50±1.35 days); small-seeded species (seed size ranged from 0.032 mg to 0.099 mg) had the lowest GP (43.63±3.68%, Figure 2B).

One-way ANOVAs indicated that seed size had significant effects on germination, which could account for 3.7% and 6.3% of the variance in GP and GT, respectively (Table 1). The linear regression analysis showed there was either no significant correlation between seed size and GP (*R^2^ = *0.005, *P* = 0.397), or no GT (*R^2^ = *0.016, *P* = 0.154) (Figure S1A, S1B). Further, two-way ANOVAs detected significant interactions between seed size and habitat, and between seed size and other life history attributes, such as dispersal mode (Table 2).

In the incomplete models, the multi-factorial ANOVAs suggested seed size accounted for 1.4% and 2.7% of the variance in GP and GT independently, respectively (Table 3, 4).

#### Dispersal mode

Seeds of adhesion-dispersed species showed the highest GP (69.84±4.60%) and the most delayed GT (18.89±1.10 days), seeds of ant-dispersed species presented the earliest GT (8.04±1.10 days), and seeds of unassisted species had the lowest GP (48.92±1.74%, Figure 2C).

One-way ANOVAs suggested that dispersal mode had significant effects on both GP and GT, which could account for 2.1% and 8.7% of the variance in GP and GT, respectively (Table 1). The multi-factorial ANOVAs suggested dispersal mode explained 0.7% and 1.5% of the variance in GP and GT independently, respectively (Table 3, 4).

#### Flowering time

One-way ANOVAs suggested that the impact of onset of flowering on GP, and the effect of duration of flowering on GT were statistically significant (Table 1). The GP of late-flowering species (59.85±2.94%) was higher than that of others. The GT of medium duration of flowering species (13.11±0.30 days) was later than that of others. Results also revealed that no linear correlations were observed, neither between GP and flowering time (*R^2^ = *0.012, *P* = 0.212) nor between GT and flowering time (*R^2^<*0.001, *P* = 0.927) (Figure S1C, S1D).

Two-way ANOVAs showed significant interaction terms for flowering time when analyzed in combination with phylogeny, life history attributes and habitat (Table 2). The multi-factorial ANOVAs suggested onset of flowering explained 0.7% of the variance in GP (Table 3), and duration of flowering explained 0.5% of the variance in GT independently (Table 4).

### Environmental correlates

#### Temperature

**Figure 3:**
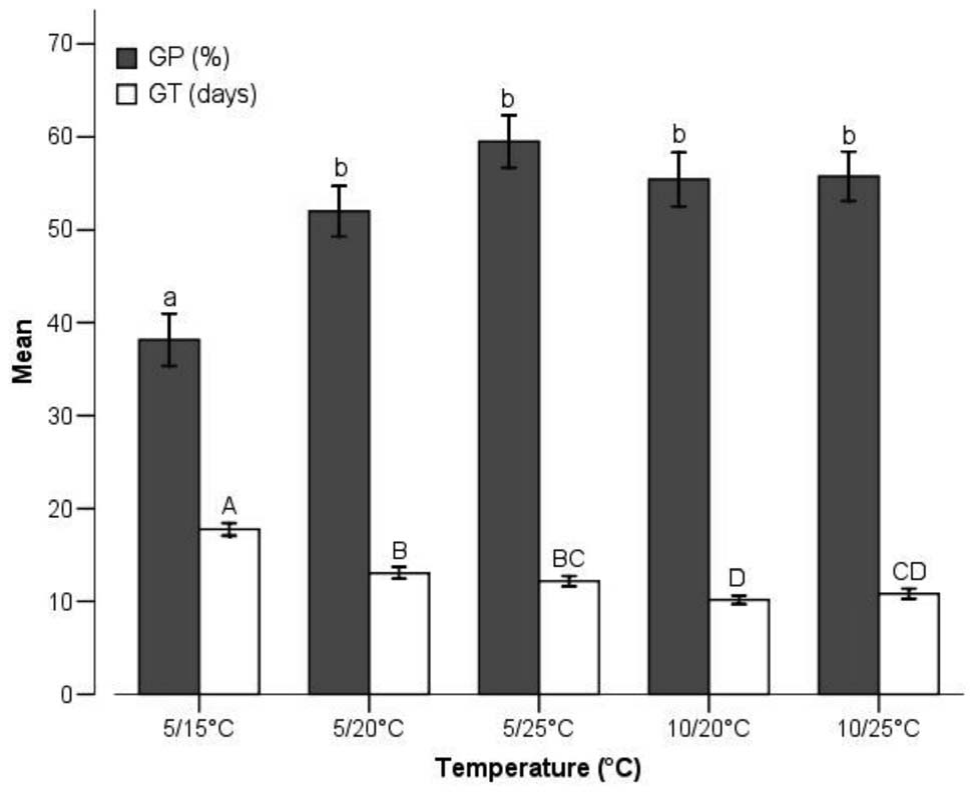
Effects of temperature on germination percentage (GP) and mean germination time (GT). Bars (mean±SE) that do not share a letter represent significantly different values at *P*<0.05 level (Turkey multiple comparison test).

**Figure 4:**
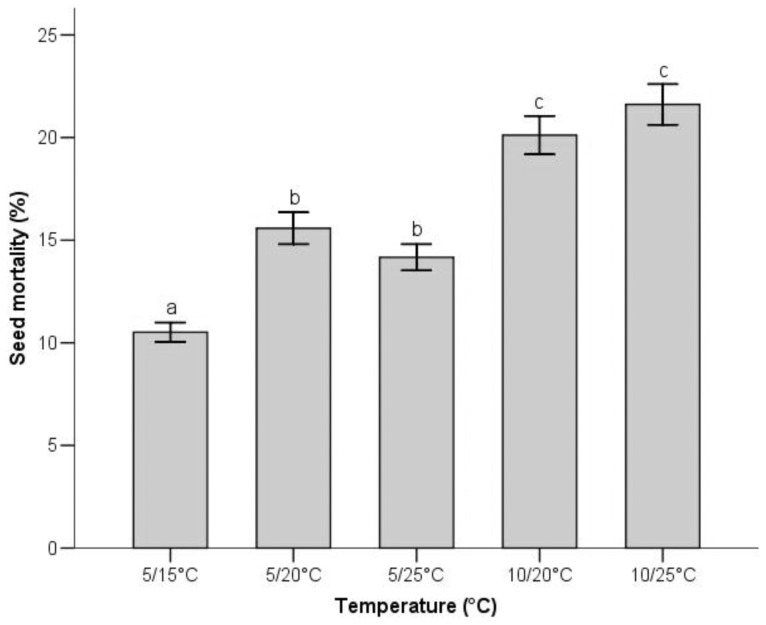
Effects of temperature treatments on seed mortality during germination. Bars (mean±SE) that do not share a letter represent significantly different values at *P*<0.05 level (Turkey multiple comparison test).

Generally, the earliest GT occurred in 10/20°C (10.15±0.47 days), and the highest GP occurred in 5/25°C (59.50±2.81%), whereas the most delayed (17.75±0.68 days) and the poorest germination (38.16±2.82%) occurred in 5/15°C (Figure 3). Tetrazolium tests revealed that most ungerminated seeds were still alive at the end of the experiments. The percentage of ungerminated but viable seeds was 51.32%, 32.43%, 26.33%, 24.46% and 22.63% at 5/15°C, 5/20°C, 5/25°C, 10/20°C and 10/25°C respectively, and the temperature treatments significantly affected the mortality of the seeds (Figure 4).

One-way ANOVAs suggested that temperature had statistically significant effects on seed germination, and explained 4.7% and 13.5% of the total variance in GP and GT, respectively (Table 1). The multi-factorial ANOVAs suggested the variance in GP and GT explained independently by temperature was 4.7% and 13.5%, respectively (Table 3, 4).

#### Habitat

Generally, seeds from bottomland displayed lower GP (44.87±3.23%) than seeds from north slope (53.99±1.55%), and seeds from south slope presented earlier GT (11.56±0.65 days) than that from other habitats (Figure 2D). One-way ANOVAs suggested that habitat had a marked effect on germination, and contributed 1.0% and 1.7% of total variance in GP and GT, respectively (Table 1). Two-way ANOVAs yielded significant interaction terms for habitat when analyzed in combination with life history attributes (Table 2). The multi-factorial ANOVAs suggested the variance in GP and GT explained independently by habitat was 0.3% and 1.0%, respectively (Table 3, 4).

## Discussion

### Phylogenetic correlates

There is a growing concern that the optimization of organisms by natural selection may be influenced or prevented by life history or phylogenetic constraints [34], and they can be used to explain variations in ecological or other traits among taxa [5], [35]. This study has confirmed that both GP and GT are phylogenetically conserved traits in an alpine/subalpine meadow community, which suggests that, despite large interspecific variation, the range of variation in these traits is limited by phylogenetic affiliation. Seed germination, like any other trait, is shaped both by the natural history of the species and by the evolutionary history of the lineage, and a large proportion of interspecific variation in germination is correlated with taxon membership, representing lineage history. Similarly, Norden et al. (2009) reported that germination delay was a phylogenetically conserved trait [6]. Thus, our findings are consistent with Norden, and further show that germination percentage is also a phylogenetically conserved trait. This is mainly due to that closely related species tend to share similar values for a given trait, typically more similar than distantly related species [36], and seed germination has coevolved with other plant traits that are directly involved in regeneration success [6].

### Life history correlates

#### Life form

In this study, we found that life form had an insignificant effect on GP,but annuals germinated significantly earlier than perennials. Similarity, Wang et al. indicated that there was no significant effect of life form on GP of 69 arid/semi-arid zone species [17], and Schippers's simulations indicated that being a non-dormant annual could be a viable strategy [37]. On the contrary, Rees suggested that in a variable environment, annuals tended to have more dormancy than perennials based on a large grass data set [9]. These different conclusions could be partially explained by the effects of life form on seed germination, which may vary over habitats or floras. In other words, species composition and life form category are different in distinct habitats or floras, and the classification principles of life form are not identical, all of these factors will affect the final results.

Moreover, there are two major reasons why annuals germinated earlier than perennials on the eastern Tibetan Plateau. For one thing, annuals are more dependent on seeds than perennials in order to be able to persist in the environment in reproduction process [14], and early-germinating species preempt biological space and gain competitive advantage over late-germinating species. For another, the short growing season is a major barrier for the survival of seedlings in alpine/subalpine meadow on the Tibetan Plateau. Therefore, in suitable conditions, rapid germination is critical for successful establishment of annuals.

#### Seed size

Seed size is an important parameter of plant fitness as it may highly influence the regeneration process of a population [38]. In our study, seeds range over three orders of magnitude in size. This likely represents multiple solutions to the same problem, for example, some plants choose to make many small seeds, and some make a few large seeds. Neither of these strategies is “better”, both may work equally well. This may explain why most of our factors (other than phylogeny) explained a very small amount of the overall variance: size does matter, but there is more than one right choice.

On the other hand, there is no accordant relationship between seed size and germination strategies in the previous studies. For example, many studies have indicated that there is a significant negative relationship between seed size and dormancy [11]. However, Wang et al. reported that germination percentages among species had a significant negative correlation with seed size [17]. Some other authors reported that seed size did not have a general effect on germination [39]. In this study, we proved that seed size had significant effects on both GP and GT (but not linear relation between germination and seed size). These different results may stem from the following reasons. Firstly, the important factors co-varying with seed size and/or seed dormancy may have been left out of consideration [11]. For example, Rees (1996) found a significant positive relationship between seed size and germination in species with specialization for dispersal but no such relationship for unspecialized seeds [15]. Secondly, seed size varies greatly among different floras and the distribution of seed size in alpine/subalpine meadows on the Tibetan Plateau is skewed to small size compared with other communities. For example, seed size of Wang's study ranged from 0.06 mg to 63.50 mg, with a mean of 10.54 mg, and 70% of the seeds were heavier than 1 mg, whereas seed size of our study ranged from 0.03 mg to 6.61 mg, with a mean of 0.98 mg, only 33% of the seeds were heavier than 1 mg. Thirdly, the environment of plants to be a better predictor of dormancy than are plant longevity and seed size combined [11]. Therefore, the differences among habitats should be considered, especially the special condition of alpine/subalpine meadow in the Tibetan Plateau.

Besides, our results revealed that species with heavier seeds had the highest GP and the most delayed GT. This is mainly because heavier seeds have larger embryos and more endosperm nutrients, which is associated with increased germination percentage [40]. Nevertheless, larger seeds would not germinate fast due to their usual opacity of thick and hard seed coats [41], and small seeds are expected to have a competitive advantage over larger seeds by having faster emergence, since small seeds have proportionally greater surface area for water absorption [42].

#### Dispersal mode

The principal models propose that presence and duration of seed germination would be correlated with seed dispersal mechanism [8], [43]. In this study the effects of dispersal mode on seed germination have been demonstrated, with adhesion-dispersed seeds germinating to higher GP than unassisted seeds (Figure 2C), which is largely for the following three reasons. Firstly, some natural enemies of seeds and seedlings (such as seed predators, parasites, herbivores and pathogens) respond to density and/or distance from the parent. Secondly, sib competition may often be more severe than competition with non-sib competition, because their patterns of resource use are probably more similar. Thirdly, some species have special microhabitat for germination and establishment. Seeds of unassisted species are most likely to experience problems noted above, thus, decrease in GP can spread these risks encountered by unassisted seeds. Our results also revealed that ant-dispersed species displayed the earliest GT. This is mainly due to avoiding being buried too deeply by ants, which may result in seeds failing to germinate.

Moreover, we found that there was strong association between dispersal mode and seed size (Table 2, 4). This correlation is frequently interpreted in terms of adaption to different lifestyles [3]. Generally speaking, small-seeded species should disperse better than large-seeded species, trading off seed size with dispersal capacity [44].

#### Flowering time

In plants, vegetative growth, flowering, seed development, dispersal, and germination typically follow in sequence with more or less overlap between the phases. So it is necessary to have a complex perspective when assessing the impact of a single phenological trait like flowering time [45]. However, there are very few studies examining the interspecific effects of flowering time on germination. Our results indicated that no linear relation existed between germination characteristics and flowering time (Figure S1C, S1D). Nevertheless, onset of flowering had a significant effect on GP and duration of flowering had a significant influence on GT, although the percentage of variance in germination explained independently by flowering time was very small (both were less than 1%, Table 3, 4). As opposed to our results, Wang et al. (2009) indicated flowering time had no marked impact on seed germination [17]. Some possible explanations for the contradiction are conceivable. Firstly, flowering phenology changes along elevation gradients, with plants at higher elevations typically flowering later than plants of the same species that grow at lower elevations [46]. Secondly, differences in flowering time are often attributable to the degree to which flowering is related by the timing of other phenophases such as seed dispersal and seed germination [47]. Thirdly, early flowering time may imply early dispersal, germination, and thus a longer period of growth available to the juvenile. But a long juvenile period, including the unfavorable season, increases the risk of mortality before reproduction. This selection pressure will involve tradeoff [48], which may lead to difficulties to determine the impact of flowering time on germination directly and simply.

Moreover, flowering periods patterns are constrained mainly by phylogenetic inertia at the family level [49]. Our results revealed that more than 50% of the late-flowering species belong to Asteraceae, and species of Asteraceae presented higher GP in alpine/subalpine meadow community (Figure 2A), which can partly explain why late-flowering species displayed higher GP.

### Environmental correlates

#### Temperature

We believe it is justified to pay close attention to temperature because it has been proven to be the most important environmental variable regulating seed dormancy and germination [10], [11]. Our results indicated that temperature had a marked effect on germination and elevated temperature would lead to a significant increase in GP and an accelerated germination compared with control (Figure 3). This is consistent to the widely accepted view. For example, Baskin and Baskin have suggested that alpine species require relatively high temperatures for germination [10]. Milbau has suggested that the germination temperature in alpine plants is relatively high in comparison with ambient temperatures [2]. On the other hand, quite a lot of seeds were ungerminated but viable in our experiments, the mean percentage of ungerminated but viable seeds was 31%. We consider that may be an adaptation to the local harsh environment. Because of the germinated seeds unable to come back formerly static status, and unable to ensure seedlings could adapt to the multivariate conditions of alpine/subalpine meadow, germination completely may eventually cause the population extinction. Spreading germination in time to disperse risk plays a key role in reproductive success [50].

More importantly, there was a significant increase in mortality rate of seeds because of temperature rise (Figure 4), and it seems that fungal attack can interpret these results. Thus, it can be inferred that the high seed mortality is likely to produce selection pressures on germination, i.e. increased germination should be selected if there is high seed mortality in high temperature, which could be one reason of good germination under a relatively high temperature environment. Furthermore, high temperature can improve and accelerate germination directly by activating enzymatic reactions occurring in the process of germination and by regulating the synthesis of hormones that affect the status of seed dormancy [10].

#### Habitat

In this study, we found that seeds from south slope presented earlier GT than that from other habitats, and seeds from bottomland displayed lower GP than seeds from north slope. There are some reasons responsible for this result. First of all, the abiotic conditions are different among north slope, south slope and bottomland due to different temperature, irradiation and water stress levels. Generally, north slope have better moisture relations, less variation in temperature, and generally less harsh conditions than south slope, so this condition appears to favor the establishment of perennial species [51]. Our results also confirmed this view. In details, the percentage of perennial species is 71% in north slope and 57% in south slope. Meanwhile, perennials germinated significantly later than annuals, which can explain why seeds from south slope displayed earlier GT than seeds from north slope. Secondly, lower GP and more delayed GT of seeds from bottomland is usually related to higher dormancy levels of seeds, because adequate moisture during seed formation is expected to result in the production of more dormant seeds than in drier conditions [52].

On the other hand, our results showed habitat had significant effects on germination (Table 1) and there were strong associations between seed size, life form, flowering time and habitat (Table 2, 3, 4), which means inherent characteristics of species may play a prominent role in evolution of germination strategies, but stochastic factors such as environmental conditions are also important selective pressures. In other words, seed germination is not only constrained by phylogenetic effects but also other factors such as environmental cues.

In our study, even the most complete ANOVAs accounted for only 22.0% of the variance in GP (Table 3) and 49.3% of the variance in GT (Table 4). Thus, we can not point out the direct cause of variation in seed germination. However, we confirm that a large proportion of interspecific variation in seed germination is correlated with taxon membership, representing lineage history. Meanwhile, selection can maintain the association between germination behavior and the environmental conditions within a lineage.

In conclusion, our results indicate that germination variation is largely dependent on phylogenetic inertia in a community. Life history factors and selection in the local environment also account for the patterns of germination in plant communities. Our results demonstrate that elevated temperature will lead to a significant increase in germination percentage and an accelerated germination. Moreover, there is a significant increase in seed mortality because of temperature rise. We infer that high seed mortality is likely to produce selection pressures on germination, which could be one reason of good germination under a relatively high temperature environment. Additionally, a significant proportion of variance in germination remains unexplained in our results, suggesting that other factors are responsible for the interspecific variation in germination displayed by alpine/subalpine species. Comprehensive studies combining community level as well as multivariate approaches are needed to enhance our understanding of the evolutionary and ecological forces shaping germination strategy.

## Supporting Information

Figure S1 **Linear relations between seed germination and life history attributes.** (A) between germination percentage (GP) and seed size; (B) between mean germination time (GT) and seed size; (C) between germination percentage (GP) and flowering time; (D) between mean germination time (GT) and flowering time. For each species, the midpoint of the flowering period is as an estimate of flowering time. (ie the midpoint of the extreme dates of a species' flowering period, given in calendar days, 1-365, starting from January 1).
(TIF)

Table S1 **The 134 alpine/subalpine species we used in the research.** The Angiosperm Phylogeny Group III(2009) was used to assign the affiliation of each species to higher levels.
(DOCX)
