# Supplementary figures and images for: Variation in Seed Germination of 134 Common Species on the Eastern Tibetan Plateau: Phylogenetic, Life History and Environmental Correlates
Source: PLoS One. 2014 Jun 3;9(6):e98601. doi: 10.1371/journal.pone.0098601 (PMC4043731; doi:10.1371/journal.pone.0098601)

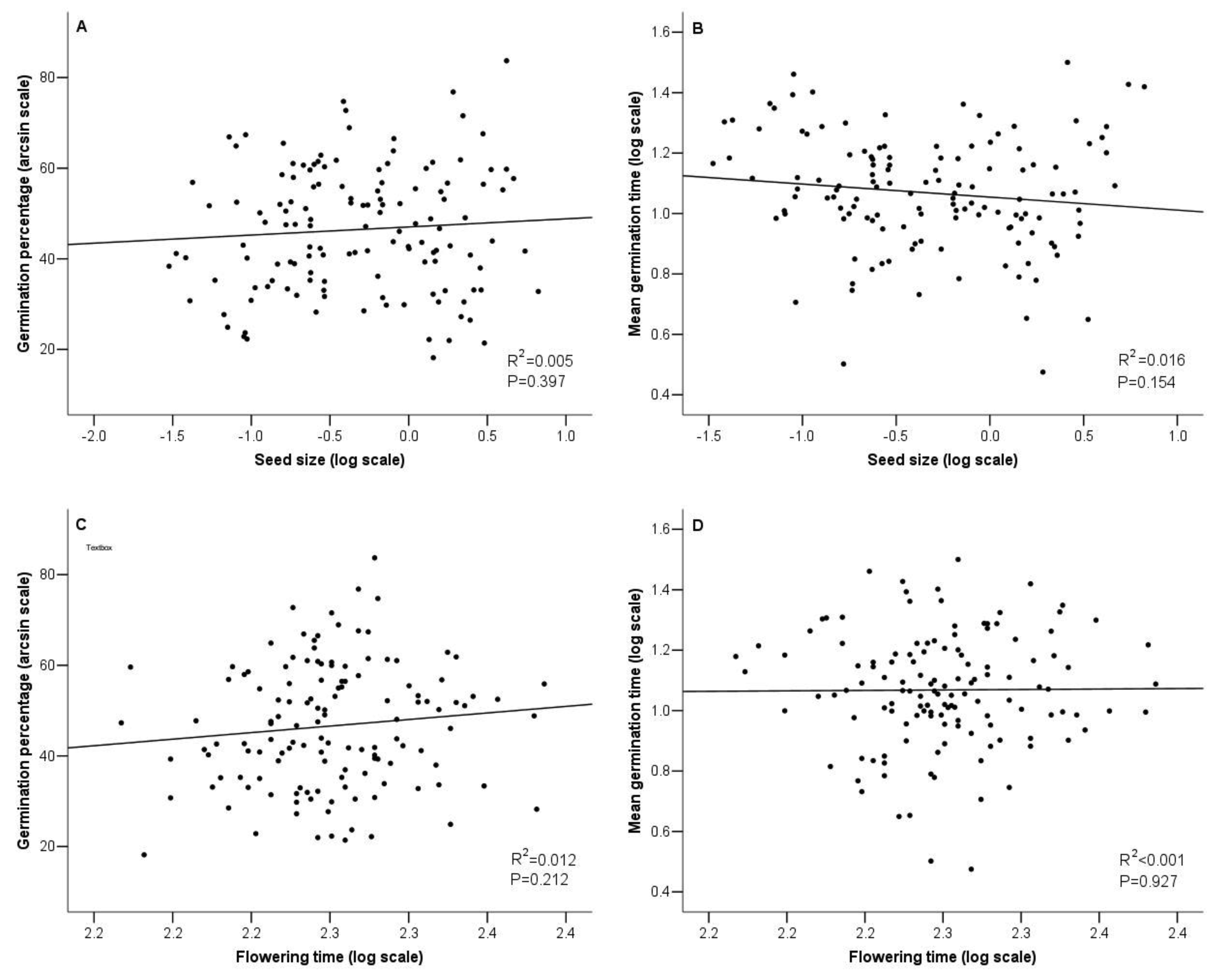

Supplement: Figure S1 — Linear relations between seed germination and life history attributes. (A) between germination percentage (GP) and seed size; (B) between mean germination time (GT) and seed size; (C) between germination percentage (GP) and flowering time; (D) between mean germination time (GT) and flowering time. For each species, the midpoint of the flowering period is as an estimate of flowering time. (ie the midpoint of the extreme dates of a species' flowering period, given in calendar days, 1-365, starting from January 1). (TIF) [file pone.0098601.s001.tif]
