# Supplementary material for: Variation in Seed Germination of 134 Common Species on the Eastern Tibetan Plateau: Phylogenetic, Life History and Environmental Correlates
Source: PLoS One. 2014 Jun 3;9(6):e98601. doi: 10.1371/journal.pone.0098601 (PMC4043731; doi:10.1371/journal.pone.0098601)
Supplement: Table S1 — The 134 alpine/subalpine species we used in the research. The Angiosperm Phylogeny Group III(2009) was used to assign the affiliation of each species to higher levels. (DOCX) [file pone.0098601.s002.docx]

**Table S1.** The 134 alpine/subalpine species we used in the research. The Angiosperm Phylogeny Group Ⅲ (2009) was used to assign the affiliation of each species to higher levels.

| Order |  |  |
| --- | --- | --- |
|  | Family |  |
|  |  | Species |
| Asterales |  |  |
|  | Asteraceae |  |
|  |  | Ajania salicifolia (Mattf.) Poljak. |
|  |  | Ajania tenuifolia (Jacq.) Tzvel. |
|  |  | Artemisia desertorum Spreng. Syst. Veg. |
|  |  | Artemisia edgeworthii Balakr. |
|  |  | Artemisia hedinii Ostenf. et Pauls. |
|  |  | Artemisia sieversiana Ehrhart ex Willd. |
|  |  | Aster flaccidus Bge. |
|  |  | Aster poliothamnus Diels |
|  |  | Aster tongolensis Franch. |
|  |  | Bidens bipinnata Linn. |
|  |  | Cremanthodium lingulatum S. W. Liu |
|  |  | Erigeron acer Linn. |
|  |  | Gerbera anandria (Linn.) Sch.-Bip. |
|  |  | Heteropappus crenatifolius (Hand.-Mazz.) Griers. |
|  |  | Heteropappus gouldii (C. E. C. Fisch.) Griers. |
|  |  | Leontopodium haplophylloides Hand.-Mazz. |
|  |  | Ligularia virgaurea (Maxim.) Mattf. |
|  |  | Paraixeris denticulata (Houtt.) Nakai |
|  |  | Parasenecio roborowskii (Maxim.) Y. L. Chen |
|  |  | Parasenecio sinicus (Ling) Y. L. Chen |
|  |  | Picris hieracioides Linn. |
|  |  | Saussurea hieracioides Hook. f. |
|  |  | Saussurea iodostegia Hance |
|  |  | Saussurea macrota Franch |
|  |  | Saussurea nigrescens Maxim. |
|  |  | Saussurea pinnatidentata Lipsch. |
|  |  | Saussurea salicifolia (Linn.) DC. |
|  |  | Senecio argunensis Turcz. |
|  |  | Senecio dubitabilis C. Jeffrey et Y. L. Chen |
|  |  | Serratula strangulata Iljin |
|  |  | Siegesbeckia pubescens Makino |
|  |  | Taraxacum maurocarpum Dahlst. |
|  | Campanulaceae |  |
|  |  | Adenophora potaninii Korsh. |
|  |  | Adenophora stenanthina (Ledeb.) Kitagawa |
|  |  | Codonopsis pilosula (Franch.) Nannf. |
|  |  | Cyananthus hookeri C. B. Cl. var. hookeri |
| Brassicales |  |  |
|  | Brassicaceae |  |
|  |  | Arabis pendula Linn. |
|  |  | Descurainia sophia (Linn.) Webb. ex Prantl |
|  |  | Draba eriopoda Turcz. |
|  |  | Eruca sativa Mill. |
|  |  | Lepidium apetalum Willd. |
|  |  | Megadenia pygmaea Maxim. |
| Caryophyllales |  |  |
|  | Amaranthaceae |  |
|  |  | Chenopodium foetidum Schrad. |
|  |  | Chenopodium glaucum Linn. |
|  |  | Chenopodium hybridum Linn. |
|  |  | Chenopodium iljinii Golosk. |
|  |  | Chenopodium prostratum Bunge |
|  |  | Corispermum tibeticum Iljin |
|  |  | Kochia scoparia (Linn.) Schrad. |
|  | Caryophyllaceae |  |
|  |  | Cerastium fontanum Baumg. subsp. triviale |
|  |  | Dianthus superbus Linn. |
|  |  | Silene aprica Turcz. ex Fisch. et Mey. var. aprica |
|  |  | Stellaria media (Linn.) Cyr. |
|  | Polygonaceae |  |
|  |  | Polygonum macrophyllum D. Don |
|  |  | Rumex crispus Linn. |
| Ericales |  |  |
|  | Primulaceae |  |
|  |  | Androsace erecta Maxim. |
|  |  | Androsace gmelinii (Gaertn.) Roem. et Schuit. |
|  |  | Pomatosace filicula Maxim. |
|  |  | Primula orbicularis Hemsl. |
|  |  | Primula stenocalyx Maxim. |
| Fabales |  |  |
|  | Fabaceae |  |
|  |  | Astragalus melilotoides Pall.* |
|  |  | Astragalus przewalskii Bunge |
|  |  | Hedysarum tanguticum B. Fedtsch. |
|  |  | Medicago lupulina Linn. |
|  |  | Oxytropis subfalcata Hance |
| Gentianales |  |  |
|  | Gentianaceae |  |
|  |  | Gentiana aristata Maxim. |
|  |  | Gentiana dahurica Fisch. |
|  |  | Gentiana pseudo-aquatica Kusnez. |
|  |  | Gentiana siphonantha Maxim. ex Kusnez. |
|  |  | Gentiana spathulifolia Maxim. ex Kusnez. |
|  |  | Gentiana straminea Maxim. |
|  |  | Gentianopsis paludosa (Hook. f.) Ma |
|  |  | Halenia elliptica D. Don |
|  |  | Lomatogonium gamosepalum (Burk.) H. Smith |
|  |  | Swertia erythrosticta Maxim. |
|  |  | Swertia handeliana H. Smith |
|  | Rubiaceae |  |
|  |  | Galium verum Linn. |
| Lamiales |  |  |
|  | Bignoniaceae |  |
|  |  | Incarvillea sinensis Lam. var. przewalskii |
|  | Lamiaceae |  |
|  |  | Ajuga lupulina Maxim. |
|  |  | Dracocephalum tanguticum Maxim. |
|  |  | Elsholtzia densa Benth. |
|  |  | Lamium amplexicaule Linn. |
|  |  | Nepeta prattii Levl. |
|  |  | Salvia roborowskii Maxim. |
|  |  | Scutellaria scordifolia Fisch. ex Schrank |
|  | Plantaginaceae |  |
|  |  | Plantago depressa Willd. |
|  |  | Plantago major Linn. |
|  | Scrophulariaceae |  |
|  |  | Euphrasia regelii Wettst. |
|  |  | Pedicularis alaschanica Maxim. |
|  |  | Pedicularis kansuensis Maxim. |
|  |  | Pedicularis polyodonta Li |
|  |  | Pedicularis verticillata Linn. |
|  |  | Veronica eriogyne H. Winkl. |
|  |  | Veronica szechuanica Batal. |
| Liliales |  |  |
|  | Liliaceae |  |
|  |  | Allium victorialis Linn. |
|  |  | Lilium pumilum DC. |
| Malpighiales |  |  |
|  | Euphorbiaceae |  |
|  |  | Euphorbia helioscopia Linn. |
|  |  | Euphorbia micractina Boiss. |
|  | Clusiaceae |  |
|  |  | Hypericum ascyron Linn. |
| Myrtales |  |  |
|  | Onagraceae |  |
|  |  | Epilobium angustifolium Linn. |
|  |  | Epilobium palustre Linn. |
| Poales |  |  |
|  | Poaceae |  |
|  |  | Agrostis hugoniana Rendle |
|  |  | Bromus japonicus Thunb. ex Murr. |
|  |  | Bromus sinensis Keng |
|  |  | Bromus tectorum Linn. |
|  |  | Deyeuxia scabrescens (Griseb.) Munro ex Duthie |
|  |  | Festuca rubra Linn. |
|  |  | Poa annua Linn. |
|  |  | Poa pratensis Linn. |
|  |  | Poa tunicata Keng ex C. Ling |
|  |  | Ptilagrostis dichotoma Keng ex Tzvel. |
|  |  | Stipa aliena Keng |
|  |  | Stipa capillacea Keng |
|  | Cyperaceae |  |
|  |  | Kobresia kansuensis Kukenth. |
|  |  | Kobresia myosuroides (Villars) Fiori |
| Ranunculales |  |  |
|  | Ranunculaceae |  |
|  |  | Aconitum gymnandrum Maxim. |
|  |  | Anemone rivularis Buch.-Ham. |
|  |  | Cimicifuga foetida Linn.* |
|  |  | Clematis tangutica (Maxim.) Korsh. |
|  |  | Delphinium grandiflorum Linn. |
|  |  | Delphinium kamaonense Huth var. glabrescens |
|  |  | Thalictrum minus Linn. |
|  |  | Thalictrum petaloideum Linn. |
|  |  | Thalictrum uncatum Maxim. |
|  |  | Trollius farreri Stapf |
|  | Papaveraceae |  |
|  |  | Meconopsis horridula Hook. |
| Rosales |  |  |
|  | Rosaceae |  |
|  |  | Geum aleppicum Jacq. |
|  |  | Potentilla multifida Linn. |
|  |  | Potentilla potaninii Wolf |
|  |  | Potentilla supina Linn. |
|  |  | Sanguisorba officinalis Linn.* |
| Saxifragales |  |  |
|  | Saxifragaceae |  |
|  |  | Saxifraga montana H. Smith |
|  | Crassulaceae |  |
|  |  | Rhodiola dumulosa (Franch.) S. H. Fu |
|  |  | Rhodiola kirilowii (Regel) Maxim. |

*Species were not included in analysis of mean germination time.
